# Phthalates and Bisphenol A: Presence in Blood Serum and Follicular Fluid of Italian Women Undergoing Assisted Reproduction Techniques

**DOI:** 10.3390/toxics8040091

**Published:** 2020-10-21

**Authors:** Donatella Paoli, Francesco Pallotti, Anna Pia Dima, Elena Albani, Carlo Alviggi, Franco Causio, Carola Conca Dioguardi, Alessandro Conforti, Rosanna Ciriminna, Gemma Fabozzi, Giuseppe Giuffrida, Roberto Gualtieri, Maria Giulia Minasi, Simona Ochetti, Valerio Pisaturo, Cinzia Racca, Laura Rienzi, Elena Sarcina, Catello Scarica, Giovanna Tomasi, Cristina Verlengia, Rita Villeggia, Federica Zullo, Andrea Lenzi, Francesco Botrè, Lucia De Santis, on behalf of the Italian Society of Embryology Reproduction and Research (SIERR)

**Affiliations:** 1 Laboratory of Seminology—Sperm Bank “Loredana Gandini”, Department of Experimental Medicine, “Sapienza”, University of Rome, 00185 Rome, Italy; francesco.pallotti@uniroma1.it (F.P.); annapia.dima@virgilio.it (A.P.D.); andrea.lenzi@uniroma1.it (A.L.); 2 Humanitas Fertility Center, Department of Gynecology, Division of Gynecology and Reproductive Medicine, Humanitas Clinical and Research Hospital—IRCCS, 20089 Milan, Italy; elena.albani@humanitas.it (E.A.); carola.conca_dioguardi@humanitas.it (C.C.D.); 3 Istituto per l’Endocrinologia e l’Oncologia sperimentale Consiglio Nazionale delle Ricerche, 80131 Naples, Italy; alviggi@unina.it; 4 Medical Centre San Luca, 70124 Bari, Italy; causio@libero.it (F.C.); dott.sarcinaelena@gmail.com (E.S.); 5 Department of Neuroscience, Reproductive Science and Odontostomatology, University of Naples Federico II, 80138 Naples, Italy; alessandro.conforti@unina.it; 6 Centro AMBRA-Palermo, 90138 Palermo, Italy; rosanna.ciriminna@gmail.com; 7 Clinica Valle Giulia, G.en.e.r.a. Centers for Reproductive Medicine, 00197 Rome, Italy; fabozzi@generaroma.it (G.F.); rienzilaura@gmail.com (L.R.); 8 CRA, Assisted Reproductive Center, 95128 Catania, Italy; giuffridag2@icloud.com (G.G.); dottoressatomasi@cragroup.it (G.T.); 9 Department Biology, University of Naples Federico II, University of Naples Federico II, 80138 Naples, Italy; roberto.gualtieri@unina.it; 10 Center for Reproductive Medicine, European Hospital-Rome, 00149 Rome, Italy; mg.minasi@gmail.com; 11 Department of Surgical Sciences, Gynecology and Obstetrics 1 Physiopathology of Reproduction and IVF Unit, S. Anna Hospital, University of Torino, 10124 Torino, Italy; sochetti@cittadellasalute.to.it (S.O.); cracca@cittadellasalute.to.it (C.R.); fedezullo4@gmail.com (F.Z.); 12 Reproductive Medicine Department, International Evangelical Hospital, 16122 Genoa, Italy; valerio@pisaturo.com; 13 Casa di cura Villa Salaria in partnership with Institut Marques, 00139 Rome, Italy; lello.scarica@gmail.com; 14 UOSD Centro PMA Sant’ Anna—ASL Roma 1, 00198 Rome, Italy; cristina.verlengia@aslroma1.it (C.V.); villerita@libero.it (R.V.); 15 Department of Experimental Medicine, “Sapienza”, University of Rome, 00153 Rome, Italy; francesco.botre@uniroma1.it; 16 Laboratorio Antidoping, Federazione Medico Sportiva Italiana, 00185 Rome, Italy; 17 IVF Unit, San Raffaele Scientific Institute Deparment Ob/Gyn, Vita-Salute University, 20132 Milan, Italy; desantis.lucia@hsr.it

**Keywords:** phthalates, BPA, follicular fluid, ART, LC-MS/MS

## Abstract

Background: folliculogenesis is a strictly regulated process that may be affected by endocrine disrupting chemicals (EDCs) through sometimes not so clear molecular mechanisms. Methods: we conducted a multicentric observational study involving six fertility centers across Italy, prospectively recruiting 122 women attending a fertility treatment. Recruited women had age ≤42 years, and normal ovarian reserve. Blood and follicular fluid samples were taken for EDCs measurement using liquid chromatography tandem mass spectrometry and each woman completed an epidemiological questionnaire. Results: The main EDCs found were monobutyl phthalate (MBP) (median blood: 8.96 ng/mL, follicular fluid 6.43 ng/mL), monoethylhexyl phthalate (MEHP) (median blood: 9.16 ng/mL, follicular fluid 7.68 ng/mL) and bisphenol A (BPA) (median blood: 1.89 ng/mL, follicular fluid 1.86 ng/mL). We found that serum MBP concentration was significantly associated with the considered area (*p* < 0.001, adj. mean: 7.61 ng/mL, 14.40 ng/mL, 13.56 ng/mL; Area 1: Milan–Turin, Area 2: Rome–Naples; Area 3: Catania–Bari, respectively) but negatively with home plastic food packaging (*p* = 0.004). Follicular MBP was associated with irregular cycles (*p* = 0.019). No association was detected between EDCs and eating habits and other clinical and epidemiological features. Conclusions: This study represents the first Italian biomonitoring of plastic EDCs in follicular fluid, laying the basis for future prospective evaluation on oocyte quality before assisted reproduction techniques (ART).

## 1. Introduction

In recent years, research has focused on the health effects of bisphenol A and phthalates. These chemical compounds are broadly used in any kind of consumer product and have been classified as endocrine disruptor chemicals (EDCs), with particular attention to possible deleterious effects on human reproduction such as a decrease of total sperm count and fertility rates [1]. Phthalates are mainly used as a plasticizer in polyvinylchloride (PVC) products and are produced through esterification with different substituents of phthalic anhydride. However, phthalates are a broad group of substances and they are classified as short-chain (low molecular weight: diethyl phthalate, di-butyl phthalate, and di-isobutyl phthalate) or long-chain (high molecular weight: benzyl-butyl phthalate, di(2-ethylhexyl) phthalate, and di-isononyl phthalate) phthalates [2]. The most common types of phthalates include di(2-ethylhexyl) phthalate (DEHP), dibutyl phthalate (DBP), dibenzyl phthalate (DBzP), diethyl phthalate (DEP) and dimethyl phthalate (DMP). Furthermore, phthalates from this last group are classified as extremely worrying substances by the REACH (registration, evaluation, authorization and restriction of chemical substances) legislation based on studies conducted on animal reproduction [3]. Bisphenol A (BPA) is mainly used to produce plastics and its derivatives have been on the market for more than 50 years. It is a key monomer in the production of epoxy resins and in the most common forms of polycarbonate, which is an almost unbreakable material [4]. However, these plastic EDCs can leak from the plastic matrix and migrate into foods or beverages, especially if these are hot (or heated) and fat rich. For this reason, human exposure to phthalates occurs especially through ingestion of contaminated water and food [5]. In the human body, diesters are first hydrolyzed to monoester by lipases and esterase in the gut, liver, lungs, and other tissues, and can undergo oxidation, second the monoesters undergo conjugation by the UDP-glucuronosyl-transferase to form the hydrophilic glucuronide conjugate, so that the conjugates are easily excreted into urine [3,6]. Similarly, absorbed BPA is rapidly object of conjugation reactions by liver enzymes with unconjugated forms comprising less than 1% of the total blood BPA. Subsequently, conjugated BPA is eliminated into urine [7]. This leads to the use of excreted conjugated phthalate metabolites and BPA as markers of exposure.

The main concerns of these EDCs involve the reproductive system and female fertility may be impaired through alterations of folliculogenesis. BPA and phthalates interference with follicular development can likely lead to infertility [8]. Phthalates levels have been associated with significantly higher risk of implantation failure in women undergoing infertility treatments and BPA levels have been associated with decreased antral follicle counts and a reduction in the oocytes number with possible links to polycystic ovary syndrome and endometriosis [1].

Several animal studies have showed that exposure to DEHP (among the most common phthalates diesters) altered sexual differentiation, inhibited androgen production, reduced testicular testosterone levels, and shortened anogenital distance (AGD) in male rats [9]. In female rats, DHEP and the monoester metabolite MEHP provoked a reduction of serum estradiol and progesterone likely mediated by inhibition of the transcription of aromatase enzyme (P450arom) that converts androgens to estrogens [1]. In vivo, DEHP was found in significantly higher concentrations in follicular fluid of women with Polycystic Ovary Syndrome (PCOS), possibly justifying alterations in steroid production and pregnancy loss after in vitro fertilization [10]. Similarly, a recent study reported a negative impact of MBP on fertilization and an association between monoethyl-phthalate (MEP) and worse—blastocyst quality [11].

Conversely, BPA possesses binding affinity for androgens (AR) and estrogens (ER) receptors, thus causing dysregulation of these steroid receptors, in addition to a thyroid hormone antagonist activity [12]. In vivo, higher concentrations of BPA were found in women with reduced ovarian reserve than controls and, in female mice, there is evidence of a possible association since low dose BPA exposure was linked to reduced levels of ovarian reserve markers [13]. Urinary BPA was also associated with worse implantation rate and a decreased number of oocytes in metaphase II [14].

Even though there is limited evidence to establish the connection between the effects by phthalates and BPA exposure and reproductive dysfunctions [8], EDCs monitoring in follicular fluid, which is in direct contact with oocytes, may offer unique opportunities to better understand the links with some female reproductive diseases. As such, the aim of this study is a first attempt of Italian biomonitoring of phthalate metabolites and bisphenol A in women undergoing medical assisted reproduction techniques in both follicular fluid (FF) and blood serum (BS).

## 2. Materials and Methods

### 2.1. Subjects, Samples Collection and Questionnaire

**Figure 1:**
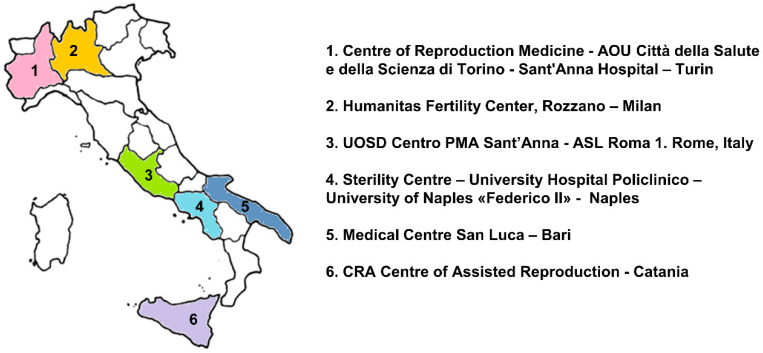
Recruiting centers.

This multicentric observational study was conducted in six fertility centers across Italy (Milan, Turin, Rome, Naples, Bari, Catania) (Figure 1). The main inclusion criteria were: women aged ≤ 42 years, undergoing oocyte retrieval for medical assisted reproduction techniques, continuously residing in the corresponding area in the last 3 years; Body Mass Index (BMI) ≤ 25 kg/m^2^; anti-Müllerian hormone (AMH) ≥ 1 ng/mL; follicle-stimulating hormone (FSH) ≤ 12 IU/L; negative medical history for PCOS, endometriosis, and no genetic pathology. All recruited women underwent ovarian stimulations and either the gonadotropin-releasing hormone (GnRH) agonist or the GnRH antagonist protocols were used in this study. On the day of oocyte retrieval, blood samples and follicular fluids were taken from each subject after overnight fasting. Blood samples were centrifuged at 2500 rpm for 10 min, frozen at −20 °C and sera were sent to the centralized laboratory for analyses together with the corresponding follicular fluids (excluding those with blood contamination). At the time of recruitment, each participant completed a self-administered questionnaire collecting information about demographics, lifestyle, medical and reproductive history, and food habits. The study was approved by the Ethical Committees from all participating centers (coordinator: Ethical Committee Policlinico Umberto I—“Sapienza” University of Rome—Protocol number 1031/17; Date of approval: 10 November 2017). All subjects signed a written informed consent form.

### 2.2. Phtalate Esters and Bisphenol a Measurement

The LC-MS/MS method here presented allows to simultaneously detect and quantify, in the same analytical run, BPA and five phthalates metabolites: mono-n-butyl phthalate (MBP), monobenzyl phthalate (MBzP), mono-ethylhexyl phthalate (MEHP), mono 2-ethyl-5-hydroxyhexyl phthalate (MEHHP) and mono-(2-ethyl-5-oxohexyl) phthalate (MEOXP). The target analytes were quantified according to the following procedure: 1 mL of follicular fluid or serum was added with 1 mL of acetonitrile for proteins precipitation, the samples were mixed for 5 min and then centrifuged for 2 min. The protein-free supernatants, obtained as described above, were prepared as follows: 1 mL of sample was added with 750 μL of potassium phosphate monobasic/sodium phosphate dibasic buffer (pH 7.0), 20 μL of the internal standard mixture (ISs: 13C4 -MEOHP and 13C12-BPA to a final concentration 10 ng/mL) and 50 μL of β-glucuronidase (Roche, Mannheim, Germany) from E. coli. Samples were then incubated for 1 h at 55 °C. After the enzymatic hydrolysis step, different extraction procedures were evaluated and compared. Between liquid/liquid (LLE) extraction using different solvents and solid phase extraction (SPE), the Oasis^®^ HLB cartridges (Waters Co., Milford, MA, USA) and the elution with methanol, was chosen. The dried fraction containing the target analytes were injected and analyzed into a high-performance liquid chromatography and tandem mass spectrometry (6495 Triple Quad LC–MS, Agilent Technologies Co., Santa Clara, CA, USA). Along with the samples, one blank and four quality controls (QCs) were run. The blank, 1mL of water, was used to assess the contamination of samples processing. The four QCs were prepared by daily spiking aliquots of working solutions (10 µg/mL) to pooled samples to obtain the desired levels (5, 25 and 100 ng/mL) to assess the accuracy and precision of the method. The limits of quantification (LOQ) were in the range of 0.3–1.25 ng/mL (Table 1), in agreement with the data reported by previous studies. For the determination of LOQ, surrogate matrices spiked with the compounds under investigation at a concentration of 5 ng/mL was used. Serial dilutions were made, and the LOQ was reported as the lowest concentration at which a compound could be quantified in all the samples tested, showing with the ion transition selected for the quantitative analysis a signal-to-noise ratio greater than 20. Calibration curves in the selected range, 0.625–100 ng/mL for both follicular fluids and serum, showed good correlation coefficients (R²) into a range of 0.998–0.999. Recoveries were of 71 ± 3%–105 ± 6%, intra-assay and inter-assay precision ≤15%. Accuracy was below 15%, for all the compounds under investigation at the four concentration levels (1.25, 5, 25 and 100 ng/mL).

### 2.3. Statistical Analysis

Continuous variables are presented as mean ± SD or median and (Interquartile range) IQR, depending on the shape of the distribution curve evaluated by the Kolmogorov–Smirnov test. Categorical variables are presented as counts and percentages and comparisons were performed by χ² test. The presence of statistically significant correlations among EDCs concentrations and other variables were evaluated using Spearman’s rank correlation test. EDCs median concentrations were compared among the different sites using the Kruskal–Wallis test with post hoc adjustment for multiple comparisons and grouped if distributions were similar between sites. As a result, for the subsequent analyses, sites were grouped in three areas: Area 1 (Milan, Turin), Area 2 (Rome, Naples), and Area 3 (Bari, Catania). For statistical analyses, food frequencies retrieved from the questionnaire were grouped into a two-level categorical variable (“high intake”: more than once a week; “low intake”: less than or equal to once a week). Differences of investigated follicular fluid and serum EDCs concentration among the considered areas were investigated using analysis of covariance, controlling for different covariates (age and BMI), as appropriate; significant results were followed up by univariate testing of significant associations and post hoc results were Bonferroni adjusted when appropriate. The probability values are two-sided; a p value less than 0.05 was considered statistically significant. All computations were carried out with Statistical Package for the Social Sciences (SPSS) 25.0 (SPSS Inc., Chicago, IL, USA).

## 3. Results

### 3.1. Demographics

Overall, 122 women undergoing assisted reproduction techniques (ART) procedures were recruited in the designated sites. Mean age of 122 women was 35.5 ± 3.7 years, while mean BMI was 21.8 ± 2.0 with no significant differences among sites. The main reasons for referring to fertility centers were male factor infertility (46.7%), female factor other than ovarian dysfunctions (33.3%), couple infertility (20%). Table 2 shows relevant demographic data from the study group.

### 3.2. EDCs Questionnaire

Over ninety percent of subjects lived in either residential areas (61.5%) or in cities outskirts (32%) homogeneously distributed among participating sites; only a few subjects lived in rural (5.7%) or industrial areas (0.8%). However, 28.1% of women stated that they previously lived (continuously, for more than 12 months) in rural areas for a mean of 15.0 ± 11.6 years. Furthermore, 32% of women lived in the vicinity of a potentially hazardous site: dump yards for solid waste (9/122; 7.3%), farms (14/122; 11.5%) or other sources of heavy air pollution (factories, port or airports) (16/122; 13.1%). These situations had comparable frequencies among sites, with the exception of Milan (only 3/29 women lived close to a potentially hazardous site) and Bari (12/20 women) (χ^2^ *p* = 0.014). Subjects recalled potential occupational contact with antiparasitic/insecticidal agents in 14.8% of cases and with other potential toxicants (glues, paints, solvents, etc.) in 28.6% of cases. Plastic food packaging was relatively widespread with no differences among geographical areas as 78.7% of subject reported its use. In particular, the reported utilization of plastic food packaging was daily for 18.9% (23/122), weekly for 45.0% (55/122) and monthly for 14.8% (18/122) of women. More than half of women, 52.4% (64/122), reported that food in plastic packaging was preserved for more than a day.

### 3.3. Food Questionnaire

**Figure 2:**
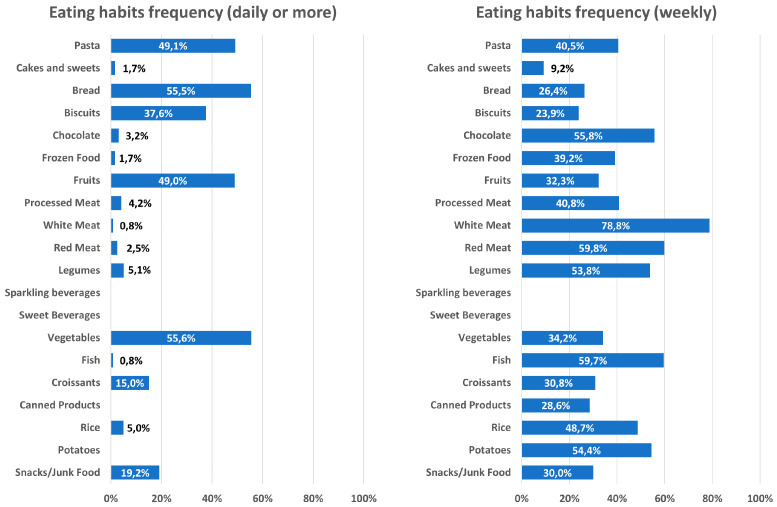
Histograms of reported frequencies of food consumption (daily or more on the left; weekly consumption on the right) for the whole cohort, based on food questionnaire answers.

**Figure 3:**
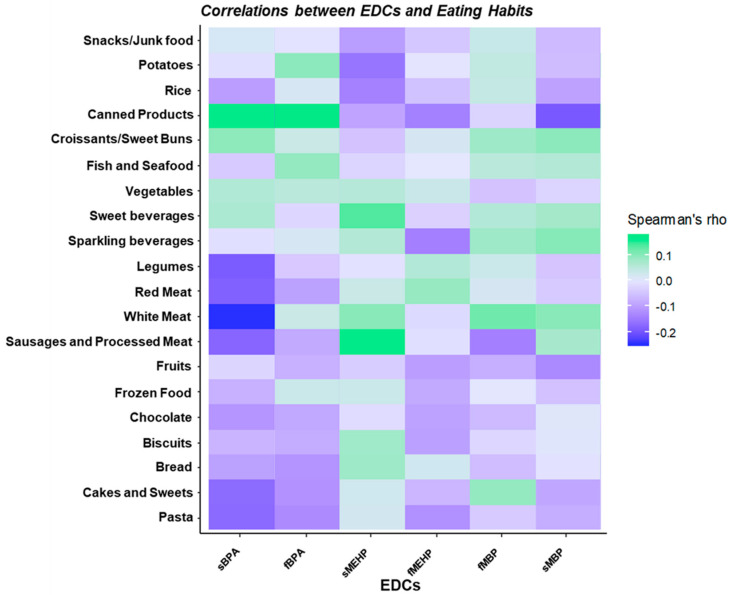
Spearman’s correlations between food consumption (high or low consumption) and serum and follicular fluid bisphenol A (BPA), monoethylhexyl phthalate (MEHP) and monobutyl phthalate (MBP). sBPA: serum BPA; fBPA: follicular fluid; sMEHP: serum MEHP; fMEHP: follicular fluid MEHP; sMBP: serum MBP; fMBP: follicular fluid MBP.

A dedicated food frequency questionnaire also investigated food habits among the recruited women. Figure 2 shows frequencies of food consumption for the whole cohort. We revealed no significant differences in reported consumption of canned/frozen food, sources of carbohydrates (bread, pasta, rice and potatoes), meat (white, red or processed), fish, fruits and sweets (cakes, chocolate, biscuits, croissants) among the three areas. Conversely, the number of women who reported to eat vegetables more than once a week was lower in centers from Area 3 (27.6% Area 3 vs. 62.5% Area 2 vs. 66.5% Area 1, χ2 *p* = 0.002) and a significantly higher number of subjects reported legumes consumption more than once a week in Area 2 compared to other areas (63.3% Area 3 vs. 82.5% Area 2 vs. 36.2% Area 1, χ2 *p* < 0.001). However, food frequencies did not correlate with either serum or follicular EDCs concentrations (Figure 3).

### 3.4. EDCs Blood and Follicular Fluid Measurement

**Figure 4:**
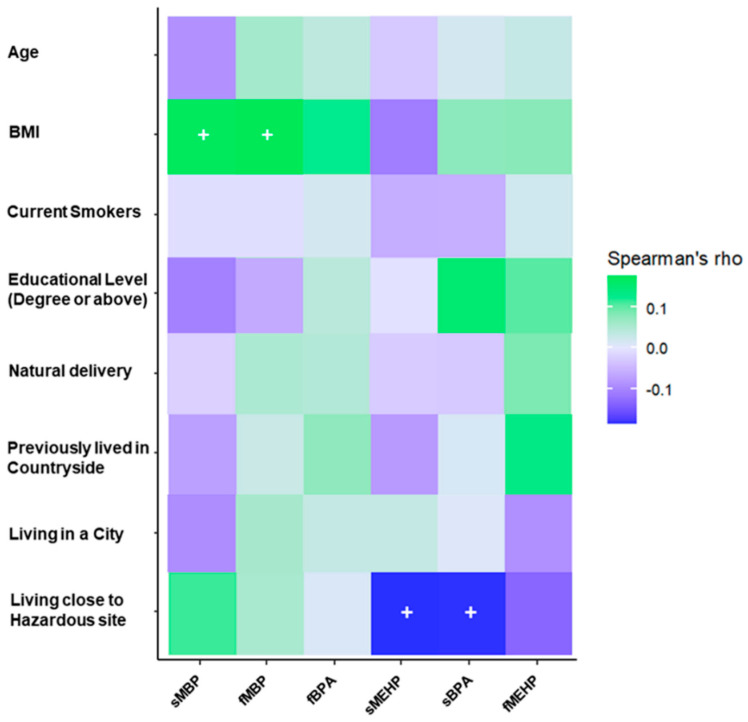
Spearman’s correlations between relevant demographic information and serum and follicular fluid BPA, MEHP and MBP. (+: *p* ≤ 0.05). sBPA: serum BPA; fBPA: follicular fluid; sMEHP: serum MEHP; fMEHP: follicular fluid MEHP; sMBP: serum MBP; fMBP: follicular fluid MBP.

The main phthalate species detected in blood and follicular fluid were monobutyl phthalate (MBP) and monoethylhexyl phthalate (MEHP), which were above the limit of quantification (LOQ) in both biological matrices in 97.5% and 77% of subjects, respectively. Bisphenol A (BPA) levels were above the LOQ in 25.4% of women. Median levels of serum and follicular EDCs and their detection rate is shown in Table 3. Serum and follicular levels of MBP and BPA were significantly correlated (Spearman’s ρ: MBP 0.567, *p* < 0.001; BPA 0.682, *p* < 0.001) (Table 4). Yet, when correcting for potential confounders (age, BMI, smoking status) only correlation between serum and follicular MBP retained significance (partial correlation: 0.293, *p* = 0.001). Figure 4 shows correlation coefficients between relevant demographic data and most represented EDCs.

Finally, when considering the three geographical areas in which the caseload was subdivided, after controlling for BMI there was a statistically significant interaction on the serum MBP and follicular MBP among the geographical areas and the utilization of plastic food packaging (F(4, 222) = 5.864, *p* < 0.001, Wilks’ Λ = 0.823, partial η^2^ = 0.093). Post hoc univariate analyses confirmed that follicular MBP was weakly associated with BMI (p = 0.035, partial η^2^ = 0.039) and showed that serum MBP was significantly associated with geographical areas (*p* < 0.001, partial η^2^ = 0.190) (Table 5) and, inversely, with the utilization of plastic food packaging (*p* = 0.004, partial η^2^ = 0.071). Moreover, univariate analyses showed that follicular but not serum MBP was significantly associated with the presence of irregular menses prior controlled ovarian stimulation (F(1, 118) = 5.619, *p* = 0.019) (Table 6). Conversely, we detected no statistically significant interaction on the combined serum MEHP and follicular MEHP levels among the geographical area and the utilization of plastic food packaging (F(4, 170) = 0.628, *p* = 0.643, Wilks’ Λ = 0.971, partial η^2^ = 0.015). Finally, regarding BPA, there was a statistically significant difference between the geographical areas on the combined serum and follicular BPA levels after controlling for BMI, F(2, 27) = 6.882, *p* = 0.004, Wilks’ Λ = 0.662, partial η^2^ = 0.338. However, univariate analyses revealed that follicular BPA levels alone were significantly associated with geographical areas (*p* = 0.001, partial η^2^ = 0.337) (Table 5).

## 4. Discussion

### 4.1. Ovarian Function and EDCs

Folliculogenesis is a strictly regulated process, necessary to ensure endocrine homeostasis and reproductive fitness in healthy women, requiring interactions of androgenic, estrogenic and gonadotropin signaling [15]. The disappointing evidence of hormonal disturbances deriving from the so-called endocrine disruptors is growing every day, underlining many different hypotheses on how EDCs can affect reproduction, from fetal reproductive organs development to menopause. In general, animal models have shown that EDCs can affect Hypothalamic-Pituitary-Gonadal (HPG) axis and ovarian function, pubertal development, and trophoblast/placental function [16]. Organochlorine compounds, pesticides and estrogenic drugs have been extensively studied, but a growing amount of data are investigating the impact of compounds such as phthalate esters and phenols (and bisphenol A in particular) [9]. These are frequently found in commonly used plastic products and, despite their short half-life, human contact is so common that exposure can be considered continuous [17]. Phthalate esters are a large group of chemicals whose DEHP and its derivate MEHP, in particular, have been linked with impairment of follicular steroidogenesis, dysregulation of primordial follicle recruitment and inhibition of antral follicle growth, peri-ovulatory follicular maturation and corpus luteum transition [12,18]. An in vitro study with cultured human granulosa cell in a MEHP containing medium showed a reduced estradiol production both as basal production and in response to gonadotropin stimulation [19]. Furthermore, the same study showed that MEHP induced estradiol inhibition was associated with a dose-dependent reduction in aromatase activity secondary to reduced aromatase mRNA transcription. The authors also proved that this inhibition is induced downwards the cAMP cascade signaling, as granulosa cell incubated with analogue 8-Br-cAMP were also affected [19]. Another in vitro study also highlighted that DEHP-induced estradiol production impairment is associated to reduced expression of both CYP19 and FSHR and with a hyperactivity of the PPAR and AhR pathways which in turn are supposed to reduce estradiol synthesis and increase its metabolism [20]. These pathways have important roles in physiological ovarian function: AhR deficient mice suffer from alterations in follicle development and have a reduced estradiol production and hindered response to gonadotropins [21]. PPARα and PPARγ are both activated by MEHP and their activation is associated with disturbances in granulosa cell steroidogenesis (reduced aromatase mRNA expression, increased CYP1B1, reduced estradiol biosynthesis and increased conversion into estrone) and differentiation [6]. Animal models have shown that BPA can interfere with antral follicle growth and granulosa/theca cells steroidogenesis by reducing expression of key enzymes (Star, Hsd3b1, Cyp17a1, Cyp19a1) as well as response to FSH stimulation, although different and even opposite effects have been observed depending on BPA dose, duration of exposure and different cellular model/animal species [12]. From a molecular point of view, BPA effects seem mediated by PPARγ through EGFR/ERK1/2 pathway [22]. In summary, all these EDCs-induced alterations may cause follicular dysregulation, failure to achieve ovulation and follicle regression [19].

### 4.2. Phthalates and BPA in Human Follicular Fluid

**Figure 5:**
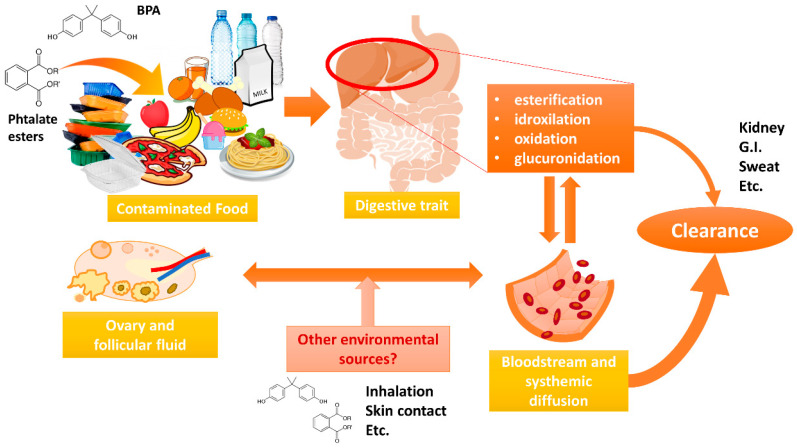
Exposure, metabolism, body circulation and elimination routes of BPA and phthalate esters.

Humans’ main source of exposure to phthalates and BPA is represented by food and reach the bloodstream through gut absorption. Like many drugs, these substances are metabolized by liver enzymes (esterification, idroxilation, oxidation, glucuronidation) increasing the compound hydrophilicity and allowing for bloodstream diffusion to other districts, the kidney where the majority of these substances are rapidly eliminated and other organs where endocrine disrupting effects may be carried out. However, several different contamination routes (skin contact, inhalation, etc.) allow a minor proportion of EDCs to reach the bloodstream and different organs (such as the ovary and follicular fluid) independently from the food exposure justifying a large variability of ECDs levels among subjects [3,5,6] (Figure 5).

Data on measurement of phthalate esters and BPA in human follicular fluid are scarce. Ikezuki et al. (2002) measured BPA levels in women from healthy women, including several who underwent in vitro fertilization (IVF) [23]. In particular, the authors detected an average BPA level of 2.4 ± 0.8 ng/mL from 36 follicular fluids. This is in contrast with another report that involved follicular fluids from five randomly selected infertile women from an IVF center: BPA was undetectable in all five samples of follicular fluid [24]. This difference could be justified by both methodological issues (the first used an ELISA measurement while the latter measured BPA through an HPLC system) and by the very low number of subjects involved by Krotz et al. In fact, in our study group we could detect follicular BPA in only about 28% of samples, in concentrations comparable to Ikezuki et al. It is possible that either quick metabolism/elimination of BPA from follicular fluid or limited exposure to BPA-containing products might contribute to the low detection rate. However, in Krotz’s study the authors detected several phthalate esters, and specifically (monoethyl-phthalate) MEP, (monoethylhexyl-phtalate) MEHP, and (mono-n-butyl-phthalate) MBP were detected in all five follicular fluids (mean 3.19 ± 2.97 ng/mL, 1.62 ± 0.59 ng/mL, 9.34 ± 3.33 ng/mL, respectively) [24]. A more recent paper reported measurement of phthalate esters in both urine and follicular fluid from Chinese women undergoing IVF, and specifically showed that most of the esters investigated were present in 70–100% of follicular fluids with exception of MBzP and MOP (43.64% and 14.55% of samples respectively); furthermore, MBP and MEHP where present in the highest concentrations (median 2.05 ng/mL and 2.80 ng/mL respectively) [25]. All these data are consistent with our findings. Indeed, we observed the almost constant presence in follicular fluid of MBP and MEHP, which also are the highest concentration phthalate metabolites found, although their median concentration is higher than Du et al. study and follicular fluid detection rates for other phthalates are somewhat lower in our study. We can also confirm the correlation between follicular and serum for MBP and BPA, but not other EDCs. Considering the confounding effects of age and, especially, BMI, only MBP retained a significant and milder correlation.

### 4.3. Possible Sources of Exposure to Phthalates and BPA

This variability might suggest that the follicular environment has some capability of metabolizing these EDCs (esterase activity), which may vary between subjects and among ethnic groups (enzymes polymorphisms), although this aspect should be deepened by further studies. Warner et al., (2019) showed that the mouse ovary can metabolize a mixture of phthalates with varying metabolizing capacity at different stages of folliculogenesis [2]. However, together with a different exposure profile deriving from a different geographical area, a different clearance rate could explain the different concentrations of phthalate esters among these studies. This might also be applicable to geographic differences we found in the different Italian areas we highlighted, as median concentrations of serum and follicular phthalates in centers from Area 1 were lower than the corresponding concentrations in the other centers (Areas 2 and 3), even when accounting for other confounders as BMI. Different exposures and, thus, EDCs follicular concentrations could also be related to work exposure, eating habits and contact to these EDCs leaking from everyday plastic products. Zota et al. (2016) investigated the presence of urinary BPA and phthalate esters in relation to fast food consumption using data from the US “National Health and Nutrition Examination Survey” (NHANES) survey, identifying an association with DEHP and DiNP in terms of both total fast food consumption (measured as total energy intake), and total fats intake; BPA instead did not appear to be significantly associated with fast food [26]. When considering of food categories, grain appeared to be associated with both DEHP and DiNP while meat only with DiNP [26]. Liao et al., (2018) detected that plastic toys, soft drinks and candies were positively associated with urinary phthalate levels in Chinese children [27]. The Euromix study also investigated associations with food habits and several endocrine disruptors, detecting significant positive associations between oily and fatty foods and DEHP urinary concentrations, while DPHP and DiBP were associated with sweets consumption. Furthermore, in this study, urinary concentration of BPA was negatively associated with dairy products intake [28]. A clear food exposure pattern is difficult to determine, nonetheless an association with fatty foods is generally accepted, based on the lipophilicity of these substances. We could not detect any significant association with food habits of the recruited women, neither when considering the consumption frequency for each food category nor when restricting the search to fatty foods (red meat, dairy, etc.) or high-intake foods. First, we should acknowledge that this discrepancy with recent literature may arise from possible recall biases, like most studies based on questionnaires. Second, food treatment and cooking may also cause significant fluctuations in the contaminating disruptor concentrations, thus influencing the food exposure pattern [29]. Surprisingly, we found an inverse association between serum MBP and the reported use of plastic food packaging. Although many phthalate esters may possibly contaminate food after leaking from the plastic matrix of these films, these are not the only possible sources of phthalates and MBP in particular. Other exposure patterns (skin, breathing) may also be considered when evaluating our results. In fact, phthalates esters are extensively used in many personal care products and cosmetics, to which women are frequently more exposed than men: several phthalate esters blood levels (MEP, MBP, MEHP) and, specifically, MBP levels are known to be higher in women of reproductive age. This is thought to be caused by frequent use of cosmetic and personal care products (perfumes, nail polish, lotions, hair products and sprays), in which these phthalates are contained in concentrations that are generally higher than those related to food contamination [18,28]. Furthermore, air exposure can also be considered when accounting total human exposure: airborne phthalates can be absorbed through breathing, but their release into the air is known to be affected by several variables like indoor temperature [30] which may ultimately determine further exposure variability among subjects. Considering that our questionnaire was mainly focused on food exposure, the high degree of variability of EDCs absorption and elimination, as well as the exposures from different sources can justify the lack of a clear correlation of the investigated foods with phthalates and/or BPA in the present study. As a concluding remark, we should highlight that a modest trend of reduction in several urinary phthalate esters (including DHEP) and BPA was detected during the last decade, reflecting the possible impact of stricter European regulations that forced substitution with other substances, thought to be less toxic [31]. This can further implicitly underline the fact that the EDCs exposure conundrum may be further complicated by different new compounds with still unclear endocrine disrupting properties that are being used and not directly measured in the present study.

## 5. Conclusions

This multicenter study represents in our knowledge the first Italian attempt to perform a bio-monitoring in different geographical areas of BPA and phthalates (MBP, MBzP, MEOXP, MEHP, MEHHP) in follicular fluid and serum from women undergoing oocyte retrieval for IVF. In particular, among phthalates we detected the presence of two species (MBP and MEHP) in the majority of women undergoing ART in both serum and follicular fluid (more than 95% and 70%, respectively), while BPA was detected in the serum of about half of subjects and in about 28% of samples of follicular fluid. Furthermore, we detected a significant direct correlation between serum and follicular concentration of MBP and BPA, despite a possible interference of BMI. Finally, multivariate analyses showed significant geographical differences in the concentration of these EDCs. Despite we determined the presence of several potential environmental sources of EDC contamination (hazardous sites close to the residence, plastic food packaging, utilization of potential toxicants, etc.), few of these were directly related to EDCs concentration in both serum and follicular fluid in our multivariate analyses, justifying the need for further investigation.

## Figures and Tables

**Table 1:** Limit of quantification (LOQ) of the investigated endocrine disruptors.

Analyte	Calibration Linearity		LOQ (ng/mL)	Recovery (%)
	Range (ng/mL)	R²		
MBP	0.625–1000	0.999	1.25	85 ± 9
MBzP	0.625–1000	0.999	0.3	78 ± 6
MEHHP	0.625–1000	0.998	0.3	90 ± 4
MOXP	0.625–1000	0.999	0.3	89 ± 7
MEHP	0.625–1000	0.999	1.25	71 ± 3
BPA	0.625–1000	0.999	1.25	105 ± 6

**Table 2:** Patients demographics. Continuous data are described as means ± standard deviations and range (in italics).

Study Group (122 Women)	
Age (years)	35.5 ± 3.7 23.0–40.0
BMI (kg/m^2^)	21.8 ± 2.0 17.0–23.8
Smokers	Current 30 (24.6%) Former 19 (15.6%)
Cigarettes/day ^a^	7.8 ± 5.4 5.0–20.0
Years of smoking ^a^	12.8 ± 5.4 3.0–20.0
Couples with previous children	12 (9.8%)
Causes of infertility	Idiopathic 24 (20.0%) Female factor 40 (33.3%) Male factor 56 (46.7%)
Education Level	Lower secondary school 26 (21.3%) Upper secondary school 46 (37.7%) Graduated or higher 50 (41.0%)
Job	Office workers 38 (31.1%) Factory/heavy workers 17 (13.9%) Freelance professionals 10 (8.2%) Merchant/shopkeepers 7 (5.7%) Housewives 13 (10.7%) Healthcare professionals 13 (10.7%) Unemployed 9 (7.4%) Teachers 13 (10.7%) Undisclosed 2 (1.6%)
Housing history	Always in a city area 87 (71.3%) Previously/currently in countryside 35 (28.7%) Residential area 75 (61.5%) City outskirts 39 (32.0%) Farming area 7 (5.7%) Industrial area 1 (0.8%)
Hazardous sites (within 500 m)	Dump yards (solid waste) 9 (7.3%) Farms 14 (11.5%) Factories, ports, airports 16 (13.1%) None 83 (68.0%)

^a^ Self-reported by current smokers only.

**Table 3:** Median and 25th–75th percentile (Q1–Q3) and detection rates of investigated endocrine disrupting chemicals (EDCs). FF: follicular fluid.

EDC	Serum Median (Q_1_–Q_3_) ng/mL	Follicular Fluid Median (Q_1_–Q_3_) ng/mL	*p* Value ^a^	Detection Rate (% above LOQ)	Simultaneous Detection Rate in Both Serum and FF (%)
MBP	8.96 (4.80–15.50)	6.43 (3.37–12.68)	<0.001	Serum: 99.2% FF: 99.2%	97.5%
MBzP	0.35 (0.30–0.56)	0.30 (0.30–0.42)	0.009	Serum: 44.3% FF: 54.1%	31.1%
MEOXP	0.34 (0.30–1.02)	0.35 (0.30–0.79)	0.904	Serum: 55.7% FF: 41.0%	27.9%
MEHHP	0.39 (0.30–0.96)	0.85 (0.30–1.75)	0.458	Serum: 58.2% FF: 36.9%	26.2%
MEHP	9.16 (4.97–17.86)	7.78 (4.12–14.90)	0.375	Serum: 95.1% FF: 79.5%	77.0%
BPA	1.89 (1.25–2.84)	1.86 (1.25–1.90)	0.287	Serum: 52.4% FF: 28.7%	25.4%

^a^ Mann–Whitney U test.

**Table 4:** Correlation coefficients (Spearman’s ρ) between serum and follicular EDCs.

EDC	MBP	MBzP	MEOXP	MEHHP	MEHP	BPA
Correlation Coefficient	0.567	0.166	0.147	0.184	0.054	0.682
*p* value	<0.001	0.680	0.107	0.043	0.555	<0.001

**Table 5:** BMI adjusted concentration means and standard error (in brackets) of relevant EDCs identified from multi- and uni-variate analyses.

	Area 1	Area 2	Area 3
Serum MBP (ng/mL)	7.61 (0.98)	14.40 ^b^ (0.96)	13.56 ^a^ (1.71)
Follicular BPA (ng/mL)	<LOQ	1.57 (0.05)	1.94 ^c^ (0.12)

^a^*p* = 0.009 vs. “Area 1” (Bonferroni adjustment for multiple comparisons). ^b^ *p* < 0.001 vs. “Area 1” (Bonferroni adjustment for multiple comparisons). ^c^ *p* = 0.009 vs. “Area 2” (Bonferroni adjustment for multiple comparisons).

**Table 6:** BMI adjusted follicular MBP concentration means and standard error (in brackets).

	Irregular Menses	Regular Menses
Follicular MBP (ng/mL)	18.83 ^a^ (2.05)	8.46 (0.92)

^a^*p* = 0.019 vs. “regular menses”.
